# Prevalence of headache at the initial stage of stroke and its relation with site of vascular involvement: A clinical study

**Published:** 2015

**Authors:** Alijan Ahmadi Aghangar, Bahareh Bazoyar, Roughayeh Mortazavi, Moazzameh Jalali

**Affiliations:** 1 Mobility Impairment Research Center Babol University of Medical Sciences, Babol, Iran.; 2 Department of Neurology, Rouhani Hospital Babol University of Medical Sciences, Babol, Iran.; 3 Neurology ward, Ayatollah Ayatollah Rouhani Hospital, Babol University of Medical Sciences, Babol, Iran.

**Keywords:** Ischemic stroke, Headache, Prevalence

## Abstract

**Background:**

Stroke is the most common neurologic disease and an important cause of morbidity and mortality. Headache is an initial presenting feature of ischemic stroke and sometimes precedes the development of stroke and thus, provides an opportunity for offering preventive measures. The aim of the present study was to determine the association of new onset headache with stroke.

**Methods:**

A total of 263 consecutive patients with stroke entered the study. Development of headache 24 have prior to admission lasting <3 days was considered the new onset headache. The intensity of headache was graded as mild, moderate and severe. Stroke was classified with respect to the localization of brain damage using magnetic resonance image (MRI). Chi-square test was applied for comparison of proportions.

**Results:**

One hundred thirty-nine males and one hundred twenty-four females with mean age of 76.4±10 (40-89) years were analyzed. Ischemic stroke involving anterior circulation was diagnosed in 210 (79%) patients and vertebrobasilar ischemia in the remaining population. Diabetes, hypertension, hyperlipidemia and coronary artery disease were observed in 36%, 52%, 38% and 42%, respectively. New onset headache was found in 49 (18.9%) patients in who 81.6% was mentioned as new onset. Six out of 9 patients with severe headache had involvement of posterior circulation, whereas in the remaining population, anterior circulation was involved.

**Conclusion:**

The findings of this study indicate no association of new onset headache with stroke. There was only a trend for severe headache toward the involvement of vertebrobasilar ischemia.

Stroke is one of the most common neurologic disorders and the third cause of death (1). Ischemic stroke either embolic or thrombotic may cause head pain, but its frequency varies widely across different studies from 7 to 65% (2-5). The entire parenchyma of the cerebrum and cerebellum and the intraparenchymal vessels are insensitive to all forms of stimulation (6, 7). Massive hemorrhage or severe edema complicating an ischemic infarct can cause headache by displacing and stretching pain sensitive intracranial structures, but the majority of ischemic infarcts are small or moderate in size and are uncomplicated by hemorrhage or severe edema, and yet are accompanied by headache. The mechanism underlying headache in stroke is not known.

However, new observations of headache in migraine may be relevant to headache in stroke. Recent studies have suggested that headache associated with ischemic cerebrovascular disease is likely vascular, and may arise from either the intracranial or the extra cranial vessels (8-10). 

The majority of patients define it without a specific character and describe it as either throbbing (17% to 54%) or continuous and non-throbbing (14% to 94%). Rarely, it may be felt as stabbing, pulsating, or having clinical features similar to intracranial hypertension. It is frequently associated with nausea (44%), vomiting (23%), and photophobia and photophobia (25%) (11, 12). Patients with large artery thrombosis develop onset headache more frequently than lacunar infarcts. Headache is also more common when there is cortical involvement (56%) than when there are subcortical infarctions (26%) (3). In clinical examination, digital compression of superficial temporal artery on the side of the headache reduces discomfort temporarily (3). It is conceivable that the final stroke in many cases is a result of a large pathological vascular process, in which headache merely serves as a warning sign of ischemic stroke (9, 11). Ischemic stroke is one of the important causes of morbidity and mortality, its treatment and rehabilitation cost too much. Headache is a common symptom in ischemic stroke. Headache may be a heralding symptom of future development of stroke. 

Therefore, awareness to prevalence and characteristics of headache provide an opportunity for preventive measures in individuals at high risk for stroke. These observations provide a rationality to do the present study. The aim of the present study was to determine the association of new onset headache with stroke.

## Methods

The study patients consisted of all cases of ischemic stroke admitted to the neurological services of 3 hospitals in Babol over a period of one year (from April 1 2011 to 31 June 2012). All eligible patients entered the study consecutively. Stroke was defined according to the criteria of the World Health Organization (13) as a relatively sudden occurrence of a focal neurologic deficit that correlates with the area of the brain supplied by the affected blood vessel. In ischemic stroke, the occlusion of a blood vessel interrupts the flow of blood to a specific region of the brain (14). Cerebral hemorrhage, transient ischemic attack, artery dissection and unconsciousness, confused or aphasic patients, patients with prior history of severe headache like tension type headache and migraine, family history of cerebrovascular disease, ischemic heart disease, contraceptive users, alcohol abusers, and cigarette smokers were not included in the present study. 

According to the International Headache Society (IHS.6.1) classification) new headache was defined as development of headache simultaneous with or in close temporal relationship to signs or other evidence of ischemic stroke confirmed by imaging and clinical symptoms and symptoms persist for more than 24 hours (15, 16).

Data were collected as to the history of headache, diabetes, hypertension, hyperlipidemia and coronary artery disease via an interview or from medical records. A headache that lasts 24 hours or longer over a 3 -days period before admission to hospital was considered new onset. The intensity of headache was graded as mild, moderate and severe. 

We investigated all patients with new onset headache that were visited by physicians or the emergency room and occurred more than 24 hours and 3-5 days before stroke onset. Blood pressure was measured and the patients were questioned about previous history of hypertension, diabetes mellitus, cardiac disease and hyperlipdemia, ECG, echocardiography, Doppler sonography of cervical vessels, brain imaging, hypercoagulable and vasculitic tests (in 40-49 years old patents). 

We classified infarcts in the anterior circulation or in the posterior circulation according to anatomical and vascular location (anterior in the territory of internal carotid artery and posterior in the vertebrobasilar territory).Chi-square test was applied for comparison of proportions. SPSS software Version 18 was used for analysis.

## Results

One hundred thirty-nine males and one hundred twenty-four females with the mean age of 76.4±10 (40-89) years were analyzed (table 1). Ischemic stroke involving anterior circulation was diagnosed in 210 (79%) patients and posterior circulation (vertebrobasilar ischemia) in the remaining subjects. Diabetes, hypertension, hyperlipidemia and coronary artery disease were observed in 36%, 52%, 38% and 42%, respectively. New onset headache was found in 49 (18.9%) patients (22 males, 18 females) and 40 out of 49 patients (81.6%) mentioned headache as new onset. Nine patients had severe headache. Six out of 9 patients with severe headache had posterior circulation involvement whereas in the remaining others, anterior circulation was involved.

**Table 1:** Characteristics of the study population with stroke

**Patient characteristics**	**N0 (%)**
Age, mean ±SD	76.4±10
Sex, female	124 (47.1)
Diabetes	95 (36)
Hypertension	137 (52)
Hyperlipidemia	100 (38)
Coronary artery disease	110 (42)
Headache	49(18.6)

## Discussion

In our study, 49 cases of 263 stroke patients (18.6%) had new onset headache. Incidence of new onset headache has been estimated to be about 4% in adult population (17, 18). In studies that addressed the prevalence of headache at the onset of stroke, the prevalence headache ranged from 7-65%) (5, 16, 19). Verdelho et al. found that headache started before stroke in 43% of subjects (5). The results of previous studies (16, 19) indicated that headache in stroke often has an organic rather than a psychogenic etiology. Although in most studies, headache is a common phenomenon in ischemic stroke, in our patients headache occurred in lower rate of stroke at 18.9% and severe new onset headache rarely happened before stroke (9 cases among our 263 patients (3.7%). Usually the stroke-related headache are unilateral, focal, and of mild to moderate severity (4, 12, 20). Whereas severe headache is usually a feature of intracranial hemorrhage, but some studies reported that this type of headache rarely happened in ischemic stroke (1, 2). On the other hand, in some studies, a significant proportion of patients (25-46%) had incapacitating pain (11) and some patients presented with thunderclap headache. In some reports, sentinel headache occurs in 10% to 43% of patients with ischemic stroke 24-72 hours before stroke onset (9, 16). Attention was drawn to the fact that sentinel headache, usually taking place prior to subarachnoid hemorrhage, is not uncommon in cerebral ischemia, occurring in 10-43% of patients (2, 4, 16). The results of this study are in agreement with other studies which most of these reports suggest that the severity of onset headache is mild to moderate (2, 4, 16), and rarely may be incapacitating (2, 4), as in our results (15.2% mild-moderate headache, 3.7% severe headache), while others indicate that headache is much more likely to be severe (45%) or moderate (39%) (11).

In our study, the mean age of ischemic stroke patients with severe new onset headache was lower than the mean age of our cases (63.3 yr in comparison 76.4 yr). There are many studies with the same results (17); in one of them, the majority of patients were younger than 72 years old (5). In our study, majority of patients suffered from bilateral headache, 7 cases (78.8%) and other 22.2% had lateralized headache. Most reports suggest that headache is lateralized, although others suggest that bilateral cranial involvement is more frequent, frontal localization is a common pattern (11). 

This study also confirms that stroke in the basilar distribution area, especially the posterior cerebral circulation (66.6%), is more often associated with headache than stroke in the carotid distribution area (34.4%). In other reports, headache is more frequent when ischemic events involve the posterior circulation (29% to 75%) (2, 3) than when they involve the anterior circulation, where headache occurs in 14% to 59% of patients (3). 

The reason for this difference is not known. The superior cerebellar artery, the proximal posterior inferior cerebellar artery, the basilar artery, and the vertebral artery were shown to be pain-sensitive (2, 11, 21-23) while the pial arteries over the superior and lateral convexities of the cerebrum and the cerebellum are insensitive to pain (2, 11, 21-24). Another possibility is that the cerebral vasculature of meninges in the posterior circulation is more heavily innervated by nociceptive afferents than the carotid circulation area. 

The stroke-related headache usually lasts for longer than 1 day. A recent study utilizing daily interviews of stroke patients developing headache has recorded a mean duration of 3.8 days (11) that was related to our results (3.1 mean duration time). It is related to a general consensus in the literature, that headache in lacunar infarction is less common (1-23%) (2, 4, 5) as none of our 9 cases with severe headache had lacunar infarcts. Although in some studies there was no relationship between headache severity and size of infarction (2, 4, 5, 11, 21), but in general, it is more severe with posterior circulation infarcts and the size of the infarction (2, 3).

Routine blood tests, hypercoagulable, vascular tests, ESR, CRP, lipid profile were in normal range in patients with severe onset headache. We did not follow our stroke patients for prognosis, but in some reports there was no association of headache in stroke onset with stroke outcome (25). There was no association of headache in stroke onset with stroke risk factors (25, 26).

In conclusion, although headache is a common symptom in ischemic stroke, severe new onset headache rarely happens. Severe headache has a trend to be associated with vertebrobasilar artery involvement.

## Notes

**Funding:** Self-funded

## Notes

**Conflict to Interest:** None declared.
